# Branching of the *p*-nitrophenol (PNP) degradation pathway in *burkholderia* sp. Strain SJ98: Evidences from genetic characterization of PNP gene cluster

**DOI:** 10.1186/2191-0855-2-30

**Published:** 2012-06-08

**Authors:** Surendra Vikram, Janmejay Pandey, Nidhi Bhalla, Gunjan Pandey, Anuradha Ghosh, Fazlurrahman Khan, Rakesh K Jain, Gajendra PS Raghava

**Affiliations:** 1 Institute of Microbial Technology (CSIR), Sector 39 A, Chandigarh 160036, India; 2 Georgia Health Sciences University, Augusta, GA, 30912, USA; 3 CSIRO- Eco Systems Sciences, Clunies Ross Street, Acton, ACT-2601, Australia; 4 Kansas State University, Manhattan, KS, 66506, USA

**Keywords:** *P*-nitrophenol, Hydroquinone dioxygenase, PNP pathway, *Burkholderia* sp SJ98

## Abstract

Aerobic microbial degradation of *p*-nitrophenol (PNP) has been classically shown to proceed via ‘Hydroquinone (HQ) pathway’ in Gram-negative bacteria, whereas in Gram-positive PNP degraders it proceed via ‘Benzenetriol (BT) pathway’. These pathways are characterized by the ring cleavage of HQ and BT as terminal aromatic intermediates respectively. Earlier reports on PNP degradation have indicated these pathways to be mutually exclusive. We report involvement of both ‘HQ’ and ‘BT’ ring cleavage pathways in PNP degradation by *Burkholderia* sp. strain SJ98. Genetic characterization of an ~41 Kb DNA fragment harboring PNP degradation gene cluster cloned and sequenced from strain SJ98 showed presence of multiple orfs including *pnpC* and *pnpD* which corresponded to previously characterized ‘benzenetriol-dioxygenase (BtD)’ and ‘maleylacetate reductase (MaR)’ respectively. This gene cluster also showed presence of *pnpE1* and *pnpE2,* which shared strong sequence identity to cognate sub-units of ‘hydroquinone dioxygenase’ (HqD). Heterologous expression and biochemical characterization ascertained the identity of PnpE1 and PnpE2. In *in vitro* assay reconstituted heterotetrameric complex of PnpE1 and PnpE2 catalyzed transformation of hydroquinone (HQ) into corresponding hydroxymuconic semialdehyde (HMS) in a substrate specific manner. Together, these results clearly establish branching of PNP degradation in strain SJ98. We propose that strain SJ98 presents a useful model system for future studies on evolution of microbial degradation of PNP.

## Introduction

**Figure 1:**
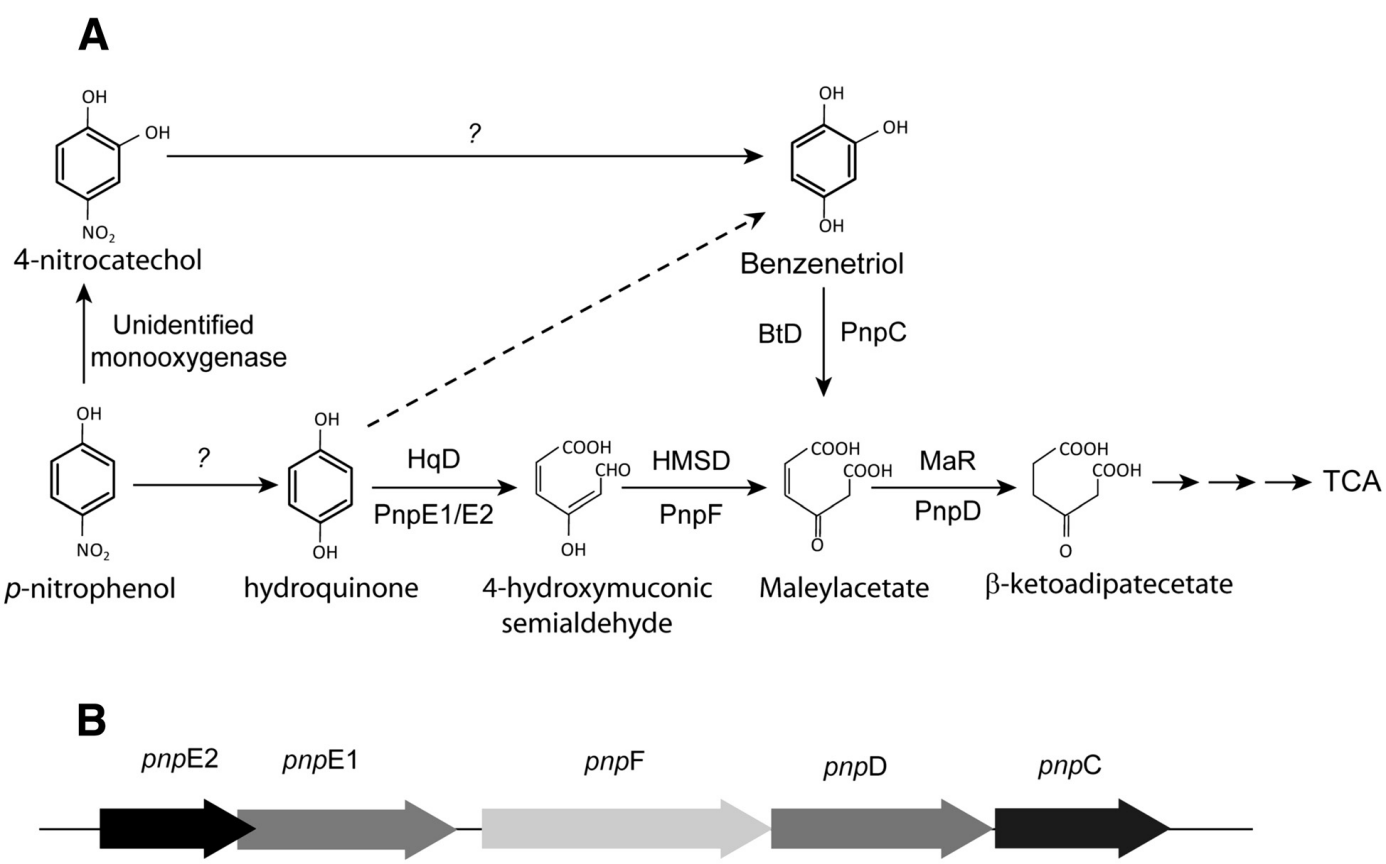
(**A**) **PNP degradation pathway in Bukholderia sp. strain SJ98. **An alternative pathway HQ to BT shown with dashed lines not present in strain SJ98. (**B**) Orientation of the ORFs found in the ~41 kb DNA fragment cloned from strain SJ98 in Supercos1 vector.

*P*-Nitrophenol (PNP) is one of the most thoroughly studied toxic environmental pollutants; it has been widely used in industries for chemical synthesis of dyes and plastics, resulting in high levels of PNP contaminations (Bhushan et al. 2000; Spain 1995). A number of Gram-positive and Gram-negative bacterial strains have been isolated and characterized for PNP degradation. Furthermore, a number of studies have shown biochemical characterization and elucidation of the catabolic pathway for PNP degradation. Results presented in these studies indicated that aerobic PNP degradation could proceed through one of the two exclusive and independent pathways (Shen et al. 2010; Chauhan et al. 2010; Kitagawa et al. 2004). PNP degradation pathway observed with Gram-positive bacteria e.g. *Arthrobacter* sp. strain JS443 (Jain et al. 1994; Perry and Zylstra 2007), *Bacillus sphaericus* strain JS905 (Kadiyala and Spain 1998), *Rhodococcus opacus* strain SAO101 (Kitagawa et al. 2004) and *Rhodococcus* sp. strain PN1 (Takeo et al. 2008) preferentially proceeds via formation of benzentriol (BT) as the terminal aromatic intermediate which undergoes ring cleavage for fission of the aromatic ring. On the other hand, Gram-negative bacteria e.g. *Moraxella* sp. strain A1 (Spain and Gibson 1991), *Pseudomonas* spp. (Liu et al. 2005; Wei et al. 2010) usually degrade PNP via formation of ‘hydroquinone’ (HQ) as the ring cleavage substrate. A few studies have indicated conversion of HQ into BT, which is then used as the terminal ring cleavage substrate in the degradation of 4-hydroxybenzoate and *p*-nitrophenol by *Candida parapsilosis* CBS604 and *Pseudomonas aeruginosa* HS-D38 respectively (Figure 1A) (Eppink et al. 2000; Zheng et al. 2009).

As variations to the above generalization, a few studies have reported alternative PNP degradation pathways. Chauhan and co-workers reported PNP degradation in *Arthrobacter protophormiae* strain RKJ100 to proceed via conversion of BT into hydroxy-benzoquinone (HBQ), which is subsequently converted into benzoquinone (BQ) and HQ (Chauhan et al., 2000). In another study, Jain et al. (1994) reported accumulation of both BT and HQ with ^14^ C trapping studies (at ~ 88% and 8% respectively) during PNP degradation by *Arthrobacter* sp. strain JS443. However, HQ could not be degraded further by strain JS443 and therefore it was suggested to be a degradation byproduct and/or dead-end degradation product (Jain et al. 1994). Genetic characterization of PNP degradation gene cluster of strain JS443 ascertained the above observation (Perry and Zylstra 2007). Another study reported PNP degradation by *Pseudomonas* sp. strain WBC-3 to selectively proceed via HQ. Noticeably, strain WBC-3 could also degraded 4-NC, wherein degradation selectively proceeds via formation of BT (Wei et al. 2010a). Based on these reports, it could be argued that microbial degradation of PNP proceeds via either BT or HQ branch of the pathway. Previously, we reported that *Burkholderia* sp. strain SJ98 (previously identified as *Ralstonia* sp. SJ98 (Samanta et al; 2000; Paul et al. 2008)); a Gram-negative bacterium, metabolizes PNP as sole source of carbon and energy. PNP induced culture of strain SJ98 degrade PNP with formation of BT and maleylacetate (MA) as major transformation intermediate, whereas 4-nitrocatechol (4-NC) and HQ are detected as transient intermediates (Chauhan et al. 2010). A 6.8 Kb DNA fragment cloned from genome of strain SJ98 showed presence of some of the genes involved in lower part of the PNP degradation pathway. Two genes viz., *pnpC* and *pnpD* showed highest sequence similarity with genes encoding for 1,2,3-benzenetriol dioxygenase (BtD) and maleylacetate reductase (MaR) respectively (Chauhan et al. 2010). Heterologous expression and biochemical characterization of these genes ascertained their identity.

In this study we have carried out further genetic characterization of PNP catabolic cluster/genes in strain SJ98. Our observations indicate co-occurrence of functional genes, encoding for enzymes involved in both ‘HQ and BT’ ring cleavage pathways of PNP degradation, within the PNP degradation gene cluster of strain SJ98. Based on these genetic evidences together with earlier report by Chauhan et al. (2010) we propose that PNP degradation in strain SJ98 shows branching and proceed via both BT and HQ branches of catabolic pathway. Similar observation for involvement of both the branches of PNP degradation pathway has been recently reported in *Pseudomonas* sp. 1–7 (Zhang et al. 2012). To the best of our knowledge, *Burkholedria* sp. strain SJ98 along with *Pseudomonas* sp.1-7 is one of the first strains showing involvement of both the branches of PNP degradation pathway.

## Materials and methods

### Bacterial strains, plasmids, chemicals, media and culture conditions

Bacterial strains, plasmids and cosmid used in this study are listed in Table 1. *Burkholderia* sp. SJ98 was grown aerobically in Luria-Bertani (LB) medium or minimal medium (MM) prepared (Samanta el al. 2000) at 30°C. *Escherichia coli* strains and recombinant clones were grown in LB medium at 37°C. Filter sterilized antibiotics were added at a final concentration of 100 μg ml^-1^ (ampicillin) or 50 μg ml^-1^ (kanamycin) wherever required. Reagents and aromatic compounds used in this study were procured from Sigma Aldrich Chemical Co. (St. Louis, MO, USA). Media components were purchased from HiMedia Laboratories Pvt. Ltd. (Mumbai, India).

**Table 1:** Bacterial strains and plasmids used in this study

**Bacterial strain/plasmid/cosmid**	**Characteristic**	**Source or reference**
**Bacterial strains**		
*Burkholderia *sp. strain SJ98	Wild type PNP degrading isolate	Lab stock and DSM = 23195
*E. coli* DH5α	Host strain for cosmid cloning vectorHost strain for GateWay entry clone	Lab stock
*E. coli *BL-21 AI	Host strain for expression vector (LacY1DE3, F_ompT hsdS, gal, dcm, ara-I)	Invitrogen Inc. CA- USA
**Plasmid/cosmid**		
SuperCos-1	Cosmid cloning vector, Dual cos sites	Agilent Technologies- Genomics. CA, USA.
pSJC88	Cosmid clone with ~41 Kb insert harboring PNP degrading gene cluster of strain SJ98	This study
pDONR-201	Gateway entry cloning vector	Invitrogen Inc. CA, USA
pDEST-17	Gateway expression vector	Invitrogen Inc. CA, USA
pDest-*pnpE1*	Expression clone with orf *pnpE1 *of strain SJ98	This study
pDest-*pnpE2*	Expression clone with orf *pnpE2 *of strain SJ98	This study

### Nucleic acid isolation and genomic library construction

Total genomic DNA of strain SJ98 was isolated and purified using Qiagen DNA Isolation Kit (Qiagen, Germany). Plasmid DNA was isolated with Qiagen Miniprep plasmid DNA Purification Kit (Qiagen, Germany) according to manufacturer’s instructions. Total cellular RNA was isolated using Promega RNeasy Miniprep Kit (Promega, USA) following manufacturers protocol. For genomic library construction, restriction digestion fragments of *Sau*3AI-digested genomic DNA of strain SJ98 were ligated to *Bam*HI-digested linearized cosmid vector SuperCos-1 (Stratagene, USA). Subsequent packaging was carried out using Gigapack III gold packaging extract (Stratagene, USA). The packaged DNA was amplified up to 10 folds and transformed into electro-competent *E. coli* DH5α cells, using GenePulser Xcell^TM^ Microbial Electroporation System (Bio-Rad Laboratories, USA). Transformed cells were spread plated onto LB- agar with ampicillin (100 μg/ml) and incubated at 37°C overnight.

### Genomic library screening and DNA sequence analyses

In order to screen the genomic library for PNP degrading gene cluster, a PCR amplicon (~540 bp) corresponding to partial BtD gene amplified from genomic DNA of strain SJ98 (Chauhan et al., 2010), was used as radio-labeled DNA probe in colony hybridization analysis. Radio labeling of the amplicon was carried out using Megaprime DNA Labeling System (GE- Healthcare, USA). Positive clones obtained in colony hybridization assay were further confirmed with colony PCR and subsequently subjected to restriction digestion analysis for determining the size of the insert in each of these. One of these clones, designated as pSJC88, with ~41 Kb insert was selected for further studies. Nucleotide sequence of the insert was determined using an ABI Model 377 DNA Sequencer and the Auto-Assembler program (Applied Biosystems, USA). Annotation of the above DNA sequence for ORF search and homology analyses of the translated nucleotide sequence was carried out using ORF finder and BLAST programs (http://www.ncbi.nlm.nih.gov/). Pair-wise alignments and identity calculations were performed using Needlman-Wunsch global alignment algorithm (http://www.ebi.ac.uk/emboss/align). Vector NTI 10.0 (Invitrogen Technologies, USA) was used to generate the graphical output report of the annotated DNA fragment.

### Cloning and expression of *pnpE1* and *pnpE2*

ORFs *pnpE1* and *pnpE2* were individually amplified with high fidelity Pfu DNA polymerase (Fermentas, USA) using primer pair listed as pnpE1F, pnpE1R and pnpE2F, pnpE2R respectively (Table 2). Cosmid DNA isolated from pSJC88 was used as the reaction template. The standard PCR reaction mix (25 μl) consisted of 100 ng cosmid DNA, 0.2 mM of each primer, 2.5 μl of 10X PCR buffer, 1 μl of 10 mM dNTPs mix, and 1.25 U of Pfu DNA polymerase (Fermentas, USA). The thermocycler program used for amplification of *pnpE1* and *pnpE2* was the following: (i) initial denaturation at 95°C for 5 min; (ii) 10 cycles of denaturation at 95°C for 1 min, primer annealing at 48°C for 30 s and fragment amplification 72°C for 1 min; (iii) followed by 25 cycles of denaturation at 95°C for 1 min, primer annealing at 63°C for 15 s and fragment amplification 72°C for 1.5 min. A final extension was performed for 10 min at 72°C. The resultant amplicons were analyzed on 1% agarose gel and later recombined with ‘Gateway Cloning Technology’ entry vector pDONR201 and destination expression vector pDEST17 (Invitrogen Technologies, USA) using BP and LR reactions respectively. The recombination cloning reactions were carried out according to the manufacturer’s recommendation. The final expression plasmids pDEST-*pnpE1* and pDEST-*pnpE2* were confirmed by nucleotide sequencing and subsequently transformed into arabinose inducible strain of *E. coli* BL-21 (Invitrogen Technologies, USA). Expression clones harboring pDEST-*pnpE1* and pDEST-*pnpE2* were grown in LB medium containing ampicillin (100 μg ml^-1^) at 30°C to an OD_600_ of ~0.4 and then induced for 8–10 hrs by adding 0.2% of Arabinose at 18°C.

**Table 2:** Oligonucleotide primers designed and used in this study

**Target ORF**	**Primer name**	**Primer sequence (5’ → 3’)**	**Application/purpose**
**PnpE1**	**pnpE1_F**	GGGGACAAGTTTGTACAAAAAAGCAGGCTTAATGGGCCGACATCTGCAT	HqD Large subunit amplification
	**pnpE1_R**	GGGGACCACTTTGTACAAGAAAGCTGGGTATTAGAACGCGACCGGATA	
**PnpE2**	**pnpE2-F**	GGGGACAAGTTTGTACAAAAAAGCAGGCTTAATGGAGACAGACATGCAA	HqD Small subunit amplification
	**pnpE2-R**	GGGGACCACTTTGTACAAGAAAGCTGGGTATTACTGGATGCAGATGTC	
**PnpE1**	**pnpE1-RT_F**	TCTACGGCTGGGTCAATTTC	HqD large subunit RT-PCR primer
	**pnpE1-RT_R**	CTTCGTTCACCCAGTCCTTC	
**PnpE2**	**pnpE2-RT_F**	CGCATTACGTGATGTCCAAC	HqD Small subunit RT-PCR primer
	**pnpE2-RT_R**	GTTTCACCGAGCCTTCGATA	
**PnpD**	**HqD_F**	AGGAGTTCATCCTGCT(G/C)(A/T)G	Partial BtD gene amplification
	**HqD_R**	CGCAC(GC)CCGAACAC(A/T)GCGTC	

### Preparation of cell extracts and purification of recombinant PnpE1 and PnpE2

500 ml culture of induced recombinant clones of pDEST-*pnpE1* and pDEST-*pnpE2* were harvested by centrifugation, washed twice with ice-cold 1X Phosphate buffered saline (PBS) and then re-suspended in 25 ml of cell lysis buffer (50 mM NaHPO_4_, 300 mM NaCl, 10% v/v glycerol and 10 mM Imidazole; pH 8.0) and lysed by ultrasonication. Soluble and insoluble fractions were separated through centrifugation at 20,000 g for 15 min. The separated fractions were tested for presence of over-expressed recombinant PnpE1 and PnpE2. Supernatant obtained from lysate of pDEST-*pnpE2* showed presence of over-expressed protein, therefore it was purified with affinity chromatography using Ni-NTA Agarose-superflow columns (Qiagen, Germany), pre-equilibrated with lysis buffer. His-tagged PnpE2 was eluted with a linear gradient of imidazole ranging from 10 mM - 200 mM in the buffer above. Thereafter, the eluted fractions were dialyzed two times with 1 l of buffer containing 50 mM NaHPO_4_, 300 mM NaCl, 10% glycerol (pH 8.0). Purified protein fractions were stored with 20% glycerol at −20°C for further use. Purity of the eluted protein samples were tested by SDS–PAGE analysis. Since, majority of the over-expressed PnpE1 was found to be present in inclusion bodies, a denaturation, dialysis, and refolding procedure was followed in order to purify PnpE1.

### Refolding and purification of PnpE1

Insoluble fractions collected from the cell lysate of *E. coli* recombinant with pDEST-*pnpE1* was resuspended in denaturing buffer (50 mM NaHPO_4_, 6 M urea, 10 mM imidazole, 300 mM NaCl, 5 mM *β*-mercaptoethanol (pH 8.0)) and incubated overnight at room temperature. Afterwards, the resuspended sample was centrifuged at 14,000 g for 30 min to separate the soluble and insoluble fractions. The soluble fraction was separated and then subjected to affinity chromatography based purification using Ni-NTA Superflow column (Qiagen, Germany) under denaturing condition according to the manufacturer’s instruction. After elution with linear gradient of imidazole in denaturing buffer, PnpE1 was diluted 100 fold in the refolding buffer (50 mM NaHPO_4_ 7.0, 10 mM imidazole, 300 mM NaCl, 5 mM BME and 10% glycerol (pH 8.0)) and allowed to refold at 4°C for 12 h. Refolded PnpE1 was again eluted through Ni-NTA Superflow beads (Qiagen, Germany) in the native purification buffer following the manufacturer’s instructions. The refolded PnpE1 was centrifuged at 14,000 × g for 30 minute to remove residual aggregate. PnpE1 was dialyzed twice with 1 l 50 mM NaHPO_4_ buffer, NaCl 300 mM, 10% Glycerol and stored at −20°C in 20% glycerol.

### Reconstitution of hydroquinone dioxygenase (HqD) with PnpE1 and PnpE2

In order to reconstitute the HqD activity, the purified recombinant proteins PnpE1 and PnpE2 were mixed in different molar ratios ranging from 3:1 to 1:3 in 1 ml of 50 mM phosphate buffer @ pH 7.0. The reconstituted protein was incubated at 30°C for 1 h and then complex protein was purified with gel filtration chromatography using Sephacryl-200 column (GE, Healthcare, USA) on an AKTA chromatography/protein purification workstation (GE Healthcare, USA). The reconstituted HqD complex(s) were subsequently analyzed for HqD activity using a spectrophotometric activity assay. The reconstituted HqD complex showing positive HqD activity (described later) was determined by comparing the elution volume in the gel filtration chromatography and its comparison with standard proteins with known molecular weights.

### HqD activity assay

The purified complexes from gel filtration chromatography described above were analyzed for HqD activity; positive HqD activity was determined spectrophotometrically by monitoring transformation of HQ used as the reaction substrate into 4-HMS (Abs_max_ at 320 nm; ε_320_ = 11.0 mM ^-1^ cm^-1^) as reported previously (Spain and Gibson 1991). The reaction mixture typically contained 50 mM NaHPO_4_ @ pH 7.5 and 100 μM FeSO_4_ and various concentrations of reconstituted HqD. Reactions were started by adding 100 μM hydroquinone prepared in dimethyl-formamide. One unit of HqD enzyme activity was defined as the amount of enzyme required for formation of 1 μmol HMS per minute. Protein concentration was measured routinely using Bradford reagent (Sigma, Germany). The gel filtration analysis carried out to determine the molecular weight of the reconstituted HqD and its purification to homogeneity was performed on a Sephacryl-200 column using Akta-prime (GE-Amersham, USA).

### Analytical methods

The HqD enzyme activity products were subjected to organic solvent extraction using equal volume of ethyl acetate thrice in neutral, acidic and alkaline pH and evaporated to dryness using a Rotavapour (Buchi, Switzerland). Extracted samples were re-suspended in 100 μl of ethyl acetate and subjected to gas chromatographic (GC) analysis using AutoSystem XL Gas chromatograph (Perkin Elmer, USA) equipped with the flame ionization detector. The thermal profile used for GC analysis was as follows: constant temperature for injector, oven and detector at 250°C, 200°C and 280°C respectively. Subsequent qualitative analysis of the reaction product was carried out with Gas chromatography–mass spectrometry (GC-MS) using Shimadzu QP2010 (Shimadzu Scientific Instruments., USA). The identity of the reaction product was ascertained by mass fragmentation pattern and its comparison with the compound database NIST62- LIB available on the GC-MS instrument.

### Induciblity of *pnpE1 and pnpE2* with RT- PCR analysis

Reverse transcription-Polymerase Chain Reaction (RT-PCR) analysis was carried out using a QIAGEN One-Step RT-PCR kit. Strain SJ98 was grown at 30°C overnight in MM with 20 mM sodium succinate. The culture was induced with 0.3 mM PNP once reached an OD_600_ of ~0.5 and was further incubated for 10–12 h at 30°C. Reverse transcriptase PCR (RT-PCR) analyses were performed to amplify internal fragments of *pnpE1* and *pnpE2* using primer pairs listed as pnpE1-RT_F, pnpE1-RT_R and pnpE2-RT_F, pnpE2-RT_R respectively (Table 2) (232 bp and 182 bp respectively) and total cellular RNA isolated as described earlier. Amplicons obtained from RT-PCR were analyzed with 1.2% agarose gel electrophoresis. Total cellular RNA isolated from cells of strain SJ98 grown on MM with 20 mM sodium succinate without PNP was used as a negative control.

### Nucleotide sequence accession number

The nucleotide and amino acid sequence data reported in this paper have been deposited to the GenBank sequence database with accession numbers JN968480 and JN968481.

## Results

### Isolation of *pnp* gene cluster from strain SJ98

In order to isolate and clone genes responsible for PNP degradation by *Burkholderia* sp. strain SJ98, a cosmid genomic library was constructed. In an earlier study the use of a partial *btd* gene fragment (~540 bp) as a probe to screen plasmid based genomic library has been described (Chauhan et al. 2010). The cosmid library was also screened using the same probe and a total of 8 positive cosmid clones (Additional file 1: Figure S1A and Additional file 1: Figure S1B). These positive clones were also verified by the amplification of *btd* specific DNA amplicon using colony PCR assay (Additional file 1: Figure S1C). Restriction digestion analysis was carried out to determine the length of the insert of the positive clones and found insert length in the range of 38 to 41 Kb (Additional file 1: Figure S1D). One of the positive clones designated as pSJC88 with insert size of ~41 Kb was selected for sequencing and further characterization.

### Sequence annotation of pSJC88

The result from sequencing of pSJC88 showed that insert of ~41 Kb harbored a 6.8 Kb *Eco*RI fragment that hybridized with BtD (specific radio labeled DNA probe). Annotation of 6.8 Kb with additional 3 Kb on either side (upstream and downstream of this fragment) showed presence of 6 orfs, with each of these being transcribed in the same direction. Two of these 6 orfs were found to be those that were previously characterized as *pnpC* and *pnpD* which encode for MaR and BtD respectively. Upstream to MaR, three other orfs viz., *pnpE1*, *pnpE2* and *pnpF* were identified. The former two showed highest sequence similarity with cognate (large and small) subunits of HqD whereas later one showed maximum sequence similarity with 4-hydroxymuconic semialdehyde dehydrogenase (4-HMSD). The BLAST search analyses indicated that *pnpE1* shared 74% sequence identity with large subunit of HqD characterized from PNP degrading *Pseudomonas* sp. strain WBC-3 (accession number ABU50916.1), while *pnpE2* had 59% sequence identity with small subunit of HqD characterized from *Pseudomonas* sp. strain WBC-3 (accession number ABU50917.1) and a hydroxyacetophenone degrading strain *Pseudomonas fluorescens* (accession number ACA50457.2). A summary of the homology searches carried out for *pnpE1*, *pnpE2* and other orfs of *pnp* gene cluster of strain SJ98 is presented in Table 3.

**Table 3:** **Amino acid sequence comparison of the ORFs identified in *pnp *** **gene cluster of *Burkholderia *** **sp. strain SJ98**

**ORF**	**Similar protein**	**Predicted function**	**% Identity**	**Score**	**E-value**	**Accession no.**
PnpC	PnpG	Hydroxyquinol dioxygenase, *Pseudomonas *sp. WBC-3	54	307	1e-102	ABU50913.1
PnpD	HapF	Maleylacetate reductase, *P. fluorescens*	60	424	1e- 146	ACA50460.1
PnpE1	PnpD	Hydroquinone dioxygenase Large subunit, *Pseudomonas *sp. WBC-3	74	536	0.0	ABU50916.1
PnpE2	PnpC	Hydroquinone dioxygenase Small subunit, *Pseudomonas *sp. WBC-3	59	195	5e-62	ABU50917.1
PnpF	PnpF	4-hydroxymuconic semialdehyde dehydrogenase, *Pseudomonas fluorescens*	73	761	0.0	ACA50459.1

### Heterologous expression of *pnp*E1 and *pnp*E2

**Figure 2:**
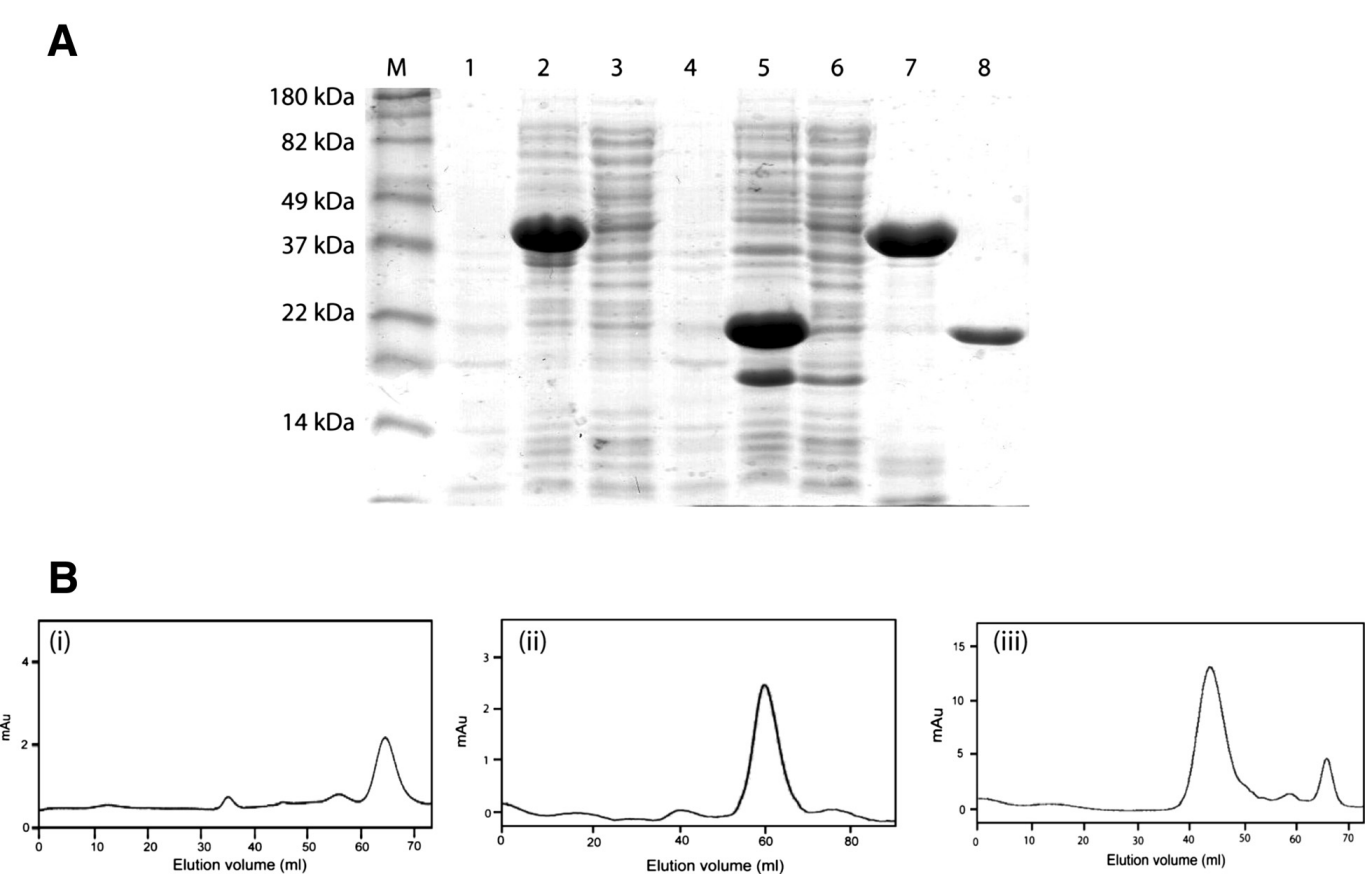
(**A**) **SDS PAGE gel showing the heterologous expression of PnpE1 and PnpE2 in E. coli BL21 AI. **The lane M: Marker; Lane 1 & 4: Uninduced supernatant of pnpE1 and pnpE2; Lane 2 & 5: Induced whole cell lysate of pnpE1 and pnpE2; Lane 3 & 6: Supernatant of pnpE1 and pnpE2 (pnpE1 not present in the supernatant); Lane 7 & 8: purified and pnpE1 (refolded) and pnpE2 respectively (**B**) Size exclusion chromatography of subunits of hydroquinone dioxygenase using sephacryl-200 (**i**) Gel filtration of PnpE1 eluted at 59.1 ml from the column and found to be a monomer of 40 kDa, (**ii**) Gel filtration of PnpE2 eluted at 65 ml and found to be a monomer of 20 kDa, (**iii**) Mixture of both the subunits found to be eluted at approximately 44 ml and the molecular weight of the hydroquinone dioxygenase predicted as approximately 120 kDa.

ORFs *pnpE1* and *pnpE2* were cloned into pDSET17 and expressed as N-terminal 6x His tagged recombinant proteins in *E. coli* BL21- AI (Invitrogen Inc. CA, USA). Cell free soluble lysates were initially examined for HqD activity without success. It was noticed that most of the over-expressed protein from clone expressing *pnpE1* was present in inclusion bodies. Therefore, insoluble fractions from this clone were subjected to urea denaturation followed by protein refolding. After refolding PnpE1 was purified with Ni-NTA affinity chromatography and analyzed with SDS PAGE analyses. SDS PAGE analysis of the purified proteins (PnpE1 and PnpE2) revealed expected molecular weight (40 kDa and 20 kDa respectively) for these His-tagged proteins (Figure 2A). Purified soluble PnpE1 and PnpE2 were tested individually for HqD activity in the spectrophotometric assay, however, no activity was observed.

### Reconstitution of active HqD complex

**Figure 3:**
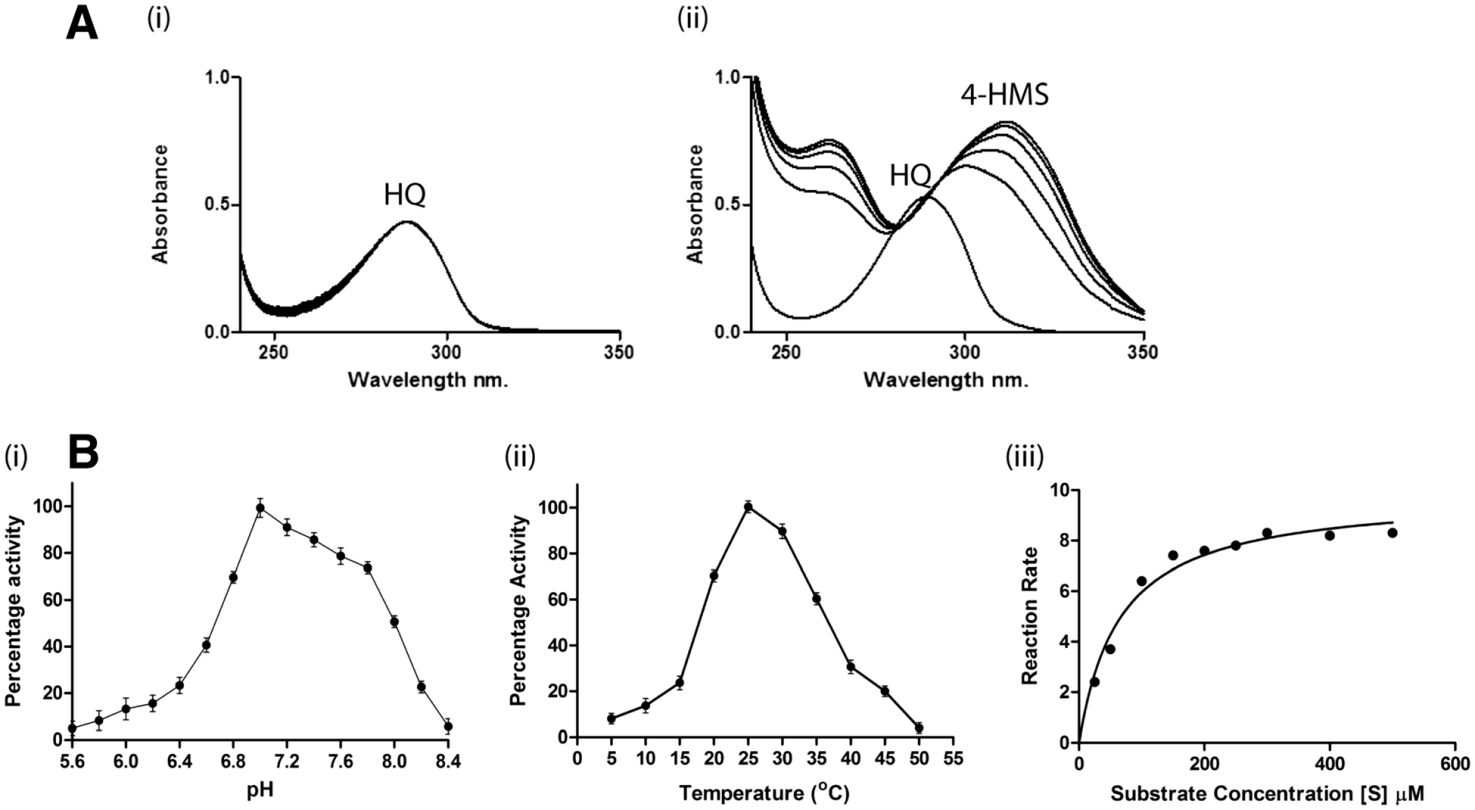
(**A**) **Enzyme activity of hydroquinone dioxygenase assayed by UV-Visible spectrophotometer. **(**i**) Negative control for the hydroquinone transformation E. coli BL21-AI (without pDEST-pnpE1 and pDEST-pnpE2) cell lysate (**ii**) Hydroquinone transformed into γ-hydroxymuconic semialdehyde and detected at the wavelength 320 nm. (**B**) The percent activity of the hydroquinone at different (**i**) pHs, (**ii**) Temperatures and (**iii**) The Michaelis-menten curve for the hydroquinone dioxygenase activity.

Since, activity assays carried out with individual subunits (PnpE1 or PnpE2) did not show any HqD activity; in subsequent activity assay, purified soluble subunits were mixed in different molecular ratios and multimeric complexes were purified over size exclusion chromatography. One of such multimeric complexes which eluted between 43–45 ml on a Sephacryl-200 column (with predicted molecular weight of ~120 kDa) was found to be positive for HqD activity in spectrophotometric assay. Figure 2B shows gel exclusion profiles of (i) PnpE2, (ii) PnpE1 and (iii) PnpE1_2_-PnpE2_2_ heterotetrameric complex. The positive HqD activity was observed with time dependent decrease in absorbance at 288 nm corresponding Ab_max_ of the reaction substrate HQ, and gradual increase in absorbance at 320 nm corresponding to 4-HMS (molar extinction coefficient = 11 mM^-1^ Cm^-1^) (Figure 3A). GC and GC-MS analyses also ascertained identity of the reaction product. The mass fragmentation pattern of reaction product obtained from the HqD activity assay showed presence of protonated pseudo molecular ion with [m/z] values of 142.04 (Additional file 1: Figure S2). The mass fragmentation pattern perfectly matched to the molecular weight of 4 − HMS.

### Catalytic properties, substrate specificity and stability of HqD from strain SJ98

Catalytic properties of HqD were routinely analyzed with spectrophotometric assay that monitors ring cleavage using HQ as reaction substrate and resulting in formation of 4 − HMS (as described above). HqD activity was observed only in presence of Fe(II) and maximum activity was measured at 100 μM FeSO_4_. HqD from strain SJ98 shows a broad pH optima ranging from pH 7.0 – 8.0 with maximum activity at pH 7.2 (Figure 3Bi). Optimal temperature for HqD activity was observed in a range of 25 – 30°C (Figure 3Bii). Kinetic analyses showed a Vmax of 9.85 ± 0.4152 μmole min^-1^ μg^-1^ and Km of 65.32 ± 10.49 μM (at pH 7.0 and 25°C) as determined from the Michaelis-Menten plot for kinetic analyses (Figure 3Biii). Reconstituted HqD catalyzed ring cleavage in a substrate specific manner with only HQ as the reaction substrate. Neither substitute hydroquinones (e.g. Chlorohydroquinone) nor other biphenolic compounds (e.g. catechol, resorcinol, chlorocatechol and nitrocatechol) were used as the reaction substrate by this enzyme (data not shown). In presence of ferrous ion chelator e.g. 2,2’-dipyridyl, the activity of HqD from strain SJ98 was completely abolished. The inactivation of enzyme activity appeared irreversible since it could not be restored by extended incubation at lower temperature with or without Fe(II). Rapid and complete inactivation of reconstituted HqD also occurred upon incubation with 100 μM hydrogen peroxide. Incubation at 40°C for 30 min and 45°C for 15 min also resulted in complete inactivation of the HqD activity.

### Induction of *pnpE1* and *pnpE2* in strain SJ98 during growth on PNP

It is important to understand whether *pnpE1* and *pnpE2* are selectively induced during growth of strain SJ98 on PNP. DNA-free total cellular RNA isolated from cells of strain SJ98, grown with and without PNP. These isolates were analyzed using RT-PCR, targeting transcripts corresponding to the above genes. In this analysis, the primer pairs were designed to amplify internal fragments corresponding to *pnpE1* and *pnpE2* transcript. RT-PCR products of expected size could be successfully amplified using RNA purified from SJ98 culture grown on PNP but not from cultures grown without PNP (Additional file 1: Figure S3). The RT- PCR products were also not observed when chemical analogues of HQ e.g. 2-chlorohydroquinone, 2-methylhydroquinone or intermediates involved in the ‘BT’ cleavage pathway e.g. 4-NC and BT were used as inducer during cell growth of strain SJ98 (data not shown). These results provide strong indication for involvement of HqD is involved in PNP degradation process.

## Discussion

Genetic characterization of gene(s) and regulatory elements involved in degradation of xenobiotic compounds is extremely important for successful development of bioremediation technologies as well as for basic understanding of biochemical and molecular mechanisms of degradation process. Hitherto, a number of studies have reported cloning and characterization of PNP degradation gene clusters. During present study, we cloned, sequenced and characterized a PNP degrading DNA fragment from *Burkholderia* sp. strain SJ98. The sequence analyses and annotation of this fragment indicated that the organization of *pnp* gene cluster in strain SJ98 is very similar to that of the *nph* gene cluster of *Arthrobacter* sp. strain JS443 (Perry and Zylstra 2007). However, the only striking difference is the presence of two orfs within *pnp* cluster of strain SJ98 that share strong sequence similarity with genes encode for cognate subunits of HqD. During earlier studies, we detected HQ as a transient metabolite in PNP degradation by strain SJ98; however, in absence of genetic information, HQ could not be ascertained as a true degradation intermediate. Instead, it was suggested that strain SJ98 may non- specifically transform PNP into HQ as a dead end product which does not contribute to the active catabolism of PNP. Similar observation has also been reported with PNP degradation by strain JS443 (Jain et al. 1994; Perry and Zylstra 2007). Results obtained during the present study and previous study with strain SJ98 clearly establish presence of a functional genes encoding for enzymes involved in both the branches of PNP degradation (viz., HqD and BtD) within the PNP degradation gene cluster of strain SJ98. Characterization of HqD carried out during present study established that this enzyme catalyzes Fe(II) dependent transformation of HQ to 4-HMS. This observation indicates that HqD from strain SJ98 belongs to ‘Ferrous ion dependent type II hydroquinone dioxygenase. Also, the active reconstituted HqD complex as predicted on the basis of the molecular weights of complex, PnpE1 and PnpE2 was found to be a heterotetrameric complex consisting of 2 molecules of both subunits. These observations are in agreement with earlier reports of HqD from other microorganisms. Ferrous ion dependent, heterotetrameric HqD complex has been previously reported to catalyze ring cleavage of HQ formed during degradation of hydroxyacetophenone (Moonen et al. 2008) and PNP (Shen et al. 2010; Zhang et al. 2009). The apparent molecular weight of reconstituted HqD from strain SJ98 (~ 120 kDa) is also within the range of molecular weights (112 kDa – 120 kDa) of other reported HqDs (Shen et al. 2010; Spain and Gibson 1991; Moonen et al. 2008; Kolvenbach et al. 2011). Noticeably, HqD from strain SJ98 also showed stringent substrate specificity in the activity assay and is selectively induced during growth of strain SJ98 on PNP; these observations clearly indicate it to be involved in transformation of HQ produced during PNP catabolism by strain SJ98. Conspicuously, stringent substrate specificity has not been observed with HqDs characterized from *Sphingomonas* sp. strain TTNP-3, *Pseudomonas fluorescens* strain ABC, *Cupriavidus necator* JMP134 and *Pseudomonas* sp. strain NyZ402 (Moonen et al. 2008; Kolvenbach et al. 2011; Yin and Zhou 2010; Wei et al. 2010b). The reason and molecular mechanism for such different behavior is unclear and is still under investigation.

Based on the results presented above, we propose orf *pnp*E1 and *pnp*E2 as genes encoding for functional cognate subunits of HqD that catalyze transformation reaction involved in HQ branch. Previously, orfs pnpC and pnpD were characterized as genes encoding for BtD and MaR that are involved in BT branch of PNP degradation pathway. Based on these results we show a graphical representation of the branching organization of *pnp* gene cluster from strain SJ98 (Figures 1A and B). Results obtained from RT-PCR analyses targeting mRNA transcripts corresponding to *pnpE1* and *pnpE2* in strain SJ98 cells grown with PNP demonstrated PNP specific induction of these genes. Previously, Chauhan et al. (2010) detected BtD and MaR specific transcripts in PNP induced cells of strain SJ98 indicating their inducible nature. Together these results present conclusive evidence for branching of PNP degradation pathway in any PNP degrading isolate. Similar observation was reported by Zhang et al. in *Pseaudomonas* sp. stain 1–7 where activity of four PNP degradation genes (*pdcDEFG*) expressed in heterologous system *in vitro*, supports the existence of HQ and BT mediated degradation pathways (Zhang et al. 2012).

Few earlier studies on genetic characterization of PNP degrading *Pseudomonads* viz., *Pseudomonas* sp. strain WBC and *Pseudomonas putida* strain DLL-E4 also reported the presence of both BtD and HqD within the same genetic locus (Shen et al. 2010; Wei et al. 2010a; Zhang et al. 2009). However, the PNP degradation in these strains proceeds only via HQ ring cleavage pathway and utilizes HqD whereas the BT cleavage enzyme BtD is only selectively utilized in 4-NC degradation (Kitagawa et al. 2004). Similar involvement for both HQ and BT branches of degradation has been reported in the degradation of 4-chlorophenol (4-CP) by *A. chlorophenolicus* A6 (Nordin et al. 2005). Nordin et al. (2005) reported involvement of two pathways but the terminal aromatic ring cleavage intermediate is the hydroxyquinol (Nordin et al. 2005). One branch of degradation of 4-CP by *A. Chlorophenolicus* A6 is proceeds via the hydroquinone and then finally into the hydroxyquinol whereas the other branch of this pathway ocuurs via the conversion of 4-CP to chlorohydroxyquinol and finally into the hydroquinone which subsequently transformed into hydroxyquinol (Nordin et al. 2005; Unell et al. 2007; Unell et al. 2009).

The annotated *pnp* gene cluster of Strain SJ98 also indicates presence of other genes required for the respective pathways. Further, studies for heterologous expression, purification, and biochemical characterization of other genes are currently underway to rigorously demonstrate involvement of both the pathways. Results obtained with biochemical characterization of purified HqD during the present study clearly demonstrate that HqD from strain SJ98 is a Fe(II) requiring, type II, heterotetrameric HqD and it represents the first enzyme of this type characterized from a member of the genus *Burkholderia*. It is actively involved in catabolic degradation of PNP by strain SJ98. Furthermore it gets selectively induced during growth of strain SJ98 on PNP. Data gathered in this study and also supported by our previous findings present conclusive evidence for co-occurrence of both ‘HQ and BT’ ring cleavage branches of PNP degradation pathway in strain SJ98. We propose *Burkholderia* sp. strain SJ98 as an important model system for further studies on evolution of PNP degradation pathways.

## Competing interests

The authors declare that they have no competing interests.

## Supplementary Material

Additional file 1 **Screening of cosmid library clones (A)colony hybridization blots were hybridized with the 540 bp partial benzenetriol dioxygenase gene.** Blot A represents preliminary round of screening and **(B)** blot B represents secondary phase of screening where only the suspected colonies were used for blotting. (+) represents the position of positive control (genomic DNA of strain SJ98). (−) represents the position of negative control (genomic DNA of E. coli DH5α). Arrows indicate hybridization signals with the probe; **(C)** Colony PCR amplification of partial (540 bp) and complete benzenetriol dioxygenase gene (818 bp) from one of the clones (Lanes 1, 2). Amplification of aldehyde dehydrogenase gene from the same clone (Lane 3). Lanes 4, 5, 6 show amplification of the same gene(s) under the same condition as with the positive control (pSJC262). Lane M represents 1 kb ladder; **(D)** The restriction pattern of cosmid clones no. 957 (Lane 2), 946 (Lane 3), 1956 (Lane 4), 1881 (Lane 5), 88 (Lane 6), 1 kb ladder (Lane M1), λ HindIII digest ladder (Lane M2). Click here for file
