# Supplementary material for: Branching of the p-nitrophenol (PNP) degradation pathway in burkholderia sp. Strain SJ98: Evidences from genetic characterization of PNP gene cluster
Source: AMB Express. 2012 Jun 8;2:30. doi: 10.1186/2191-0855-2-30 (PMC3485097; doi:10.1186/2191-0855-2-30)
Supplement: Additional file 1 — Screening of cosmid library clones (A)colony hybridization blots were hybridized with the 540 bp partial benzenetriol dioxygenase gene. Blot A represents preliminary round of screening and (B) blot B represents secondary phase of screening where only the suspected colonies were used for blotting. (+) represents the position of positive control (genomic DNA of strain SJ98). (−) represents the position of negative control (genomic DNA of E. coli DH5α). Arrows indicate hybridization signals with the probe; (C) Colony PCR amplification of partial (540 bp) and complete benzenetriol dioxygenase gene (818 bp) from one of the clones (Lanes 1, 2). Amplification of aldehyde dehydrogenase gene from the same clone (Lane 3). Lanes 4, 5, 6 show amplification of the same gene(s) under the same condition as with the positive control (pSJC262). Lane M represents 1 kb ladder; (D) The restriction pattern of cosmid clones no. 957 (Lane 2), 946 (Lane 3), 1956 (Lane 4), 1881 (Lane 5), 88 (Lane 6), 1 kb ladder (Lane M1), λ HindIII digest ladder (Lane M2). [file 2191-0855-2-30-S1.doc]

**Supplementary Material**

**Cloning and analysis of ‘hydroquinone dioxygenase’ from the *p-* nitrophenol (PNP) degrading gene cluster of *Burkholderia* sp. SJ98**

Surendra Vikram^1^, Janmejay Pandey^1,2a^, Nidhi Bhalla^1^, Gunjan Pandey^1,2b^, Anuradha Ghosh^1,2c^, Fazlurrahman Khan^1^, R. K. Jain^1,†^ and G. P. S. Raghava^1,*^

**^1^**Institute of Microbial Technology (CSIR), Sector 39 A, Chandigah (160036) India

**Present adderess**

^2a^Georgia Health Sciences University, Augusta, Georgia (30912) United States

^2b^CSIRO- Eco Systems, Black Mountain Laboratories, ACT Canberra (2601) Australia

**^2c^**Kansas State University, Manhattan, Kansas (66506) United States

**^†^**Deceased*** Corresponding Author:** Institute of Microbial Technology, Sector-39A, Chandigarh, India Phone: +91-172-2690557, Fax: +91-172-2690632; E-mail: raghava@imtech.res.in


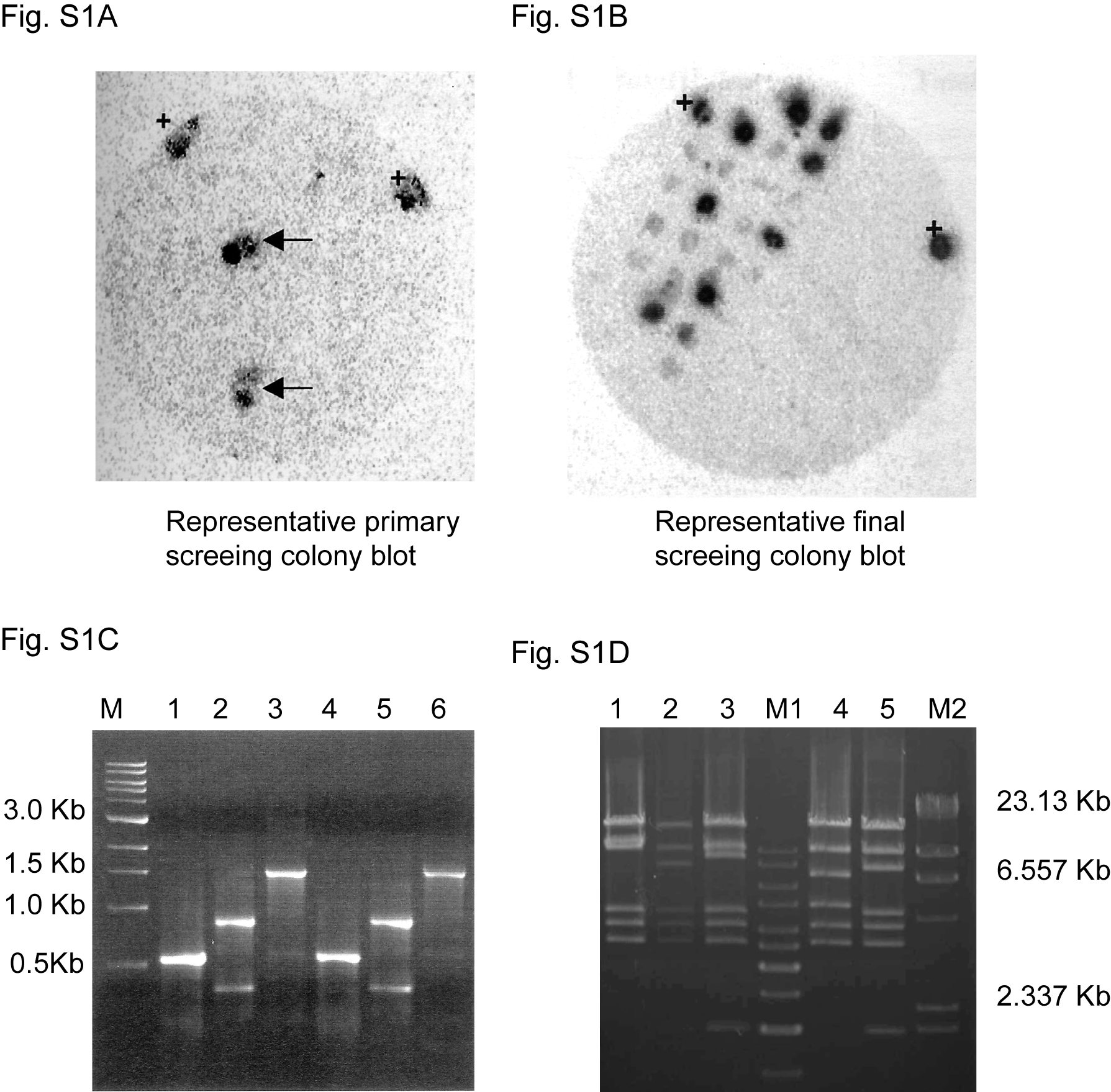


Screening of cosmid library clones **(A)** colony hybridization blots were hybridized with the 540 bp partial benzenetriol dioxygenase gene. Blot A represents preliminary round of screening and **(B)** blot B represents secondary phase of screening where only the suspected colonies were used for blotting. (+) represents the position of positive control (genomic DNA of strain SJ98). (-) represents the position of negative control (genomic DNA of E. coli DH5α). Arrows indicate hybridization signals with the probe; **(C)** Colony PCR amplification of partial (540 bp) and complete benzenetriol dioxygenase gene (818 bp) from one of the clones (Lanes 1, 2). Amplification of aldehyde dehydrogenase gene from the same clone (Lane 3). Lanes 4, 5, 6 show amplification of the same gene(s) under the same condition as with the positive control (pSJC262). Lane M represents 1 kb ladder; **(D)** The restriction pattern of cosmid clones no. 957 (Lane 2), 946 (Lane 3), 1956 (Lane 4), 1881 (Lane 5), 88 (Lane 6), 1 kb ladder (Lane M1), λ HindIII digest ladder (Lane M2).

**
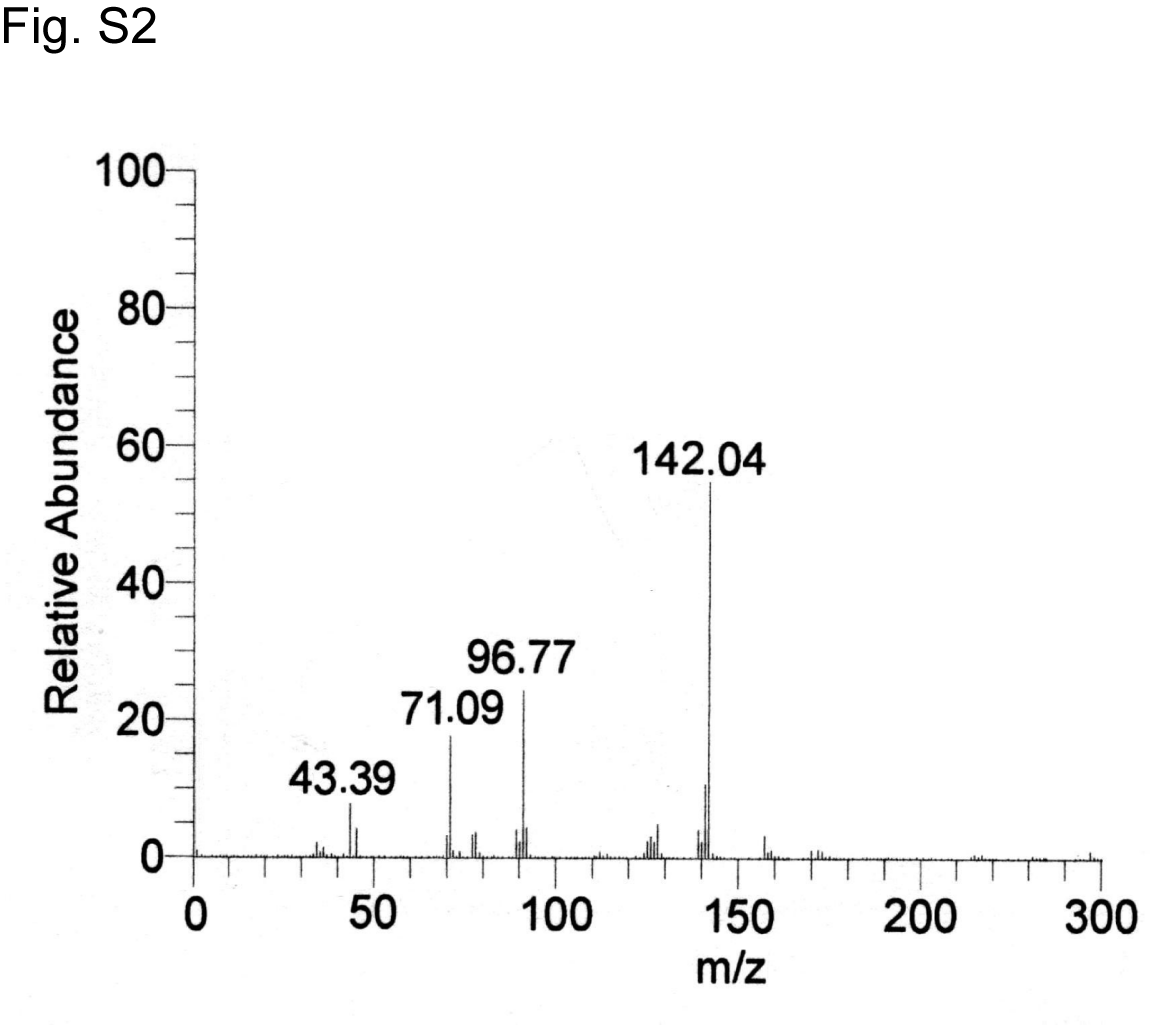
**

Mass fragmentation pattern of transformed product (γ-hydroxymuconic semialdehyde) of hydroquinone.

**
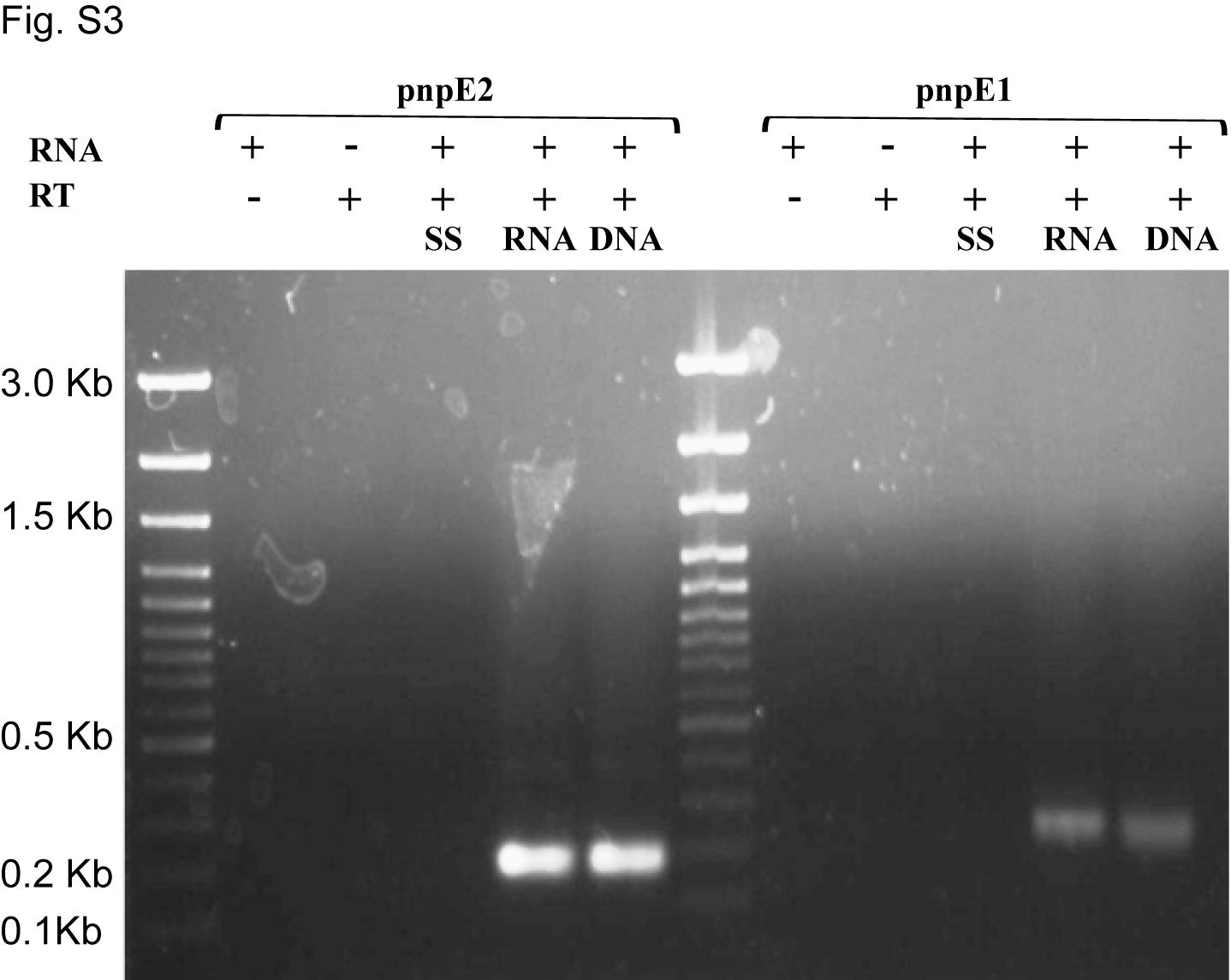
**

Agarose gel electrophoresis analysis of reverse transcriptase PCR products of pnpE1and pnpE2 of strain SJ98. Lane M represents 100bp DNA marker. Lanes 1, 2 and 6, 7 represent the controls used either without RNA template or reverse transcriptase enzyme for pnpE2 and pnpE1 respectively. Lanes 3 & 8 represent the results of the experiments with cells of strain SJ98 grown on sodium succinate (SS). Lanes 4 and 9 represent the results of the experiments with cells of strain SJ98 grown only on PNP. Lanes 5 and 10 represent amplification of pnpE2 and pnpE1 when genomic DNA was used as template.
